# Reconstruction of the Coracoclavicular Ligament Complex Utilizing an All-Suture Tape Cerclage Technique

**DOI:** 10.1016/j.eats.2024.103184

**Published:** 2024-08-10

**Authors:** William A. Ranson, Laura Thurber, Akshar V. Patel, Christoph Schroen, Carl M. Cirino, Patrick J. Denard, Paul J. Cagle

**Affiliations:** a Department of Orthopaedic Surgery, Icahn School of Medicine at Mount Sinai, New York, New York, U.S.A.; b Department of Orthopaedic Surgery, Hospital for Special Surgery, New York, New York, U.S.A.; c Department of Orthopaedics and Rehabilitation, Oregon Health & Science University, Portland, Oregon, U.S.A.

## Abstract

Current techniques for the operative management of acromioclavicular joint separation injuries are plagued by a high rate of postoperative complications. Loss of fixation has been the most difficult challenge to overcome, with a recent meta-analysis finding postoperative subluxation in over 20% of cases. No gold-standard surgical treatment has been established despite over 100 unique procedures having been described in the literature. All-suture fixation techniques have shown promise but were previously limited by issues inherent to the properties of the available suture materials. Recently, however, a modern suture product with unique properties has been made commercially available. The senior authors sought to adapt this material to fixation of injuries of the coracoclavicular ligament complex. In this Technical Note, we present an all-suture tape cerclage technique for the fixation of high-grade acromioclavicular separation injuries (Rockwood types IV, V, and VI). By controlling the ultimate knot stack position through the directionality of suture passage, this technique negates the risk of subsequent hardware irritation. Further, by avoiding the formation of coracoid tunnels, the risk of iatrogenic coracoid fracture is minimized. Importantly, this technique is simple and reproducible while also minimizing material requirements and cost.

Injuries to the coracoclavicular (CC) ligament complex occur across a spectrum of severity, with resultant acromioclavicular (AC) joint subluxation determined by the post-traumatic integrity of the AC and CC ligaments. Operative intervention is generally recommended for patients with high-grade injuries corresponding to Rockwood types IV, V, and VI. There is no gold-standard surgical technique in the treatment of these injuries, however, and more than 100 unique procedures have been described.^1^ Irrespective of the technique used, AC joint reconstruction is plagued by a high rate of loss of reduction postoperatively. A recent meta-analysis of 58 studies examined the pooled results of 1,704 patients treated with a variety of surgical techniques and found the overall rate of fixation failure to be 20.8%.^2^

Suture tape cerclage products have recently become commercially available. Developer-reported advantages include an ultimate load to failure considerably greater than previously available synthetic suture materials. Such products have garnered significant interest in the treatment of injuries involving the CC ligament complex.^3^^,^^4^ Appreciating the unique physical properties of this recently available suture material and recognizing an opportunity for its application, the senior authors (P.J.C. and P.J.D.) sought to develop a reliable, reproducible technique for surgical reduction and stabilization of the CC interval with an all-suture tape construct.

This Technical Note introduces a recently developed technique for fixing high-grade AC separation injuries that negates the risk of both symptomatic hardware and iatrogenic coracoid fracture.^5^ The technique minimizes material requirements, is reproducible, and has proven reliable in maintaining postoperative reduction of the CC interval.

## Surgical Technique

**Fig 1:**
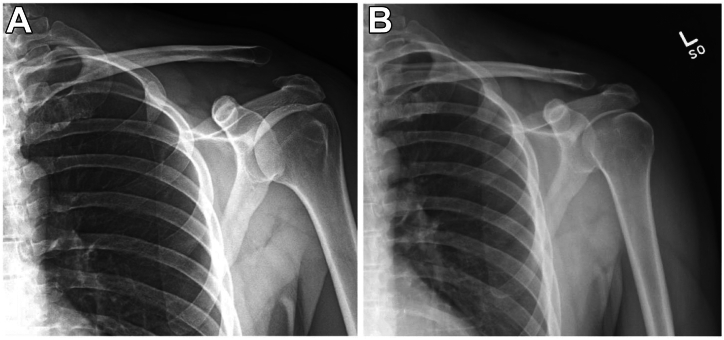
(A) Preoperative plain radiograph of the left shoulder demonstrating a type V acromioclavicular joint separation injury. (B) One-week postoperative plain radiograph of the left shoulder of the same patient demonstrating successful reduction of the acromioclavicular joint and restoration of the coracoclavicular interval.

A detailed audiovisual description of the surgical technique can be seen in Video 1 while the associated pre- and postoperative radiographs are presented in Figure 1.

In the preoperative holding area, a single shot block is performed or an interscalene anesthetic infusion catheter placed by the anesthesia team. In the operating room, the patient is placed in the beach-chair position with the operative limb secured to the Spider Limb Positioner (Smith & Nephew). The coracoid is palpated and marked. A vertical line is drawn from the coracoid to the superior aspect of the clavicle. The skin is incised and the subcutaneous fat is dissected in line with the incision until the deltotrapezial fascia is identified.

**Fig 2:**
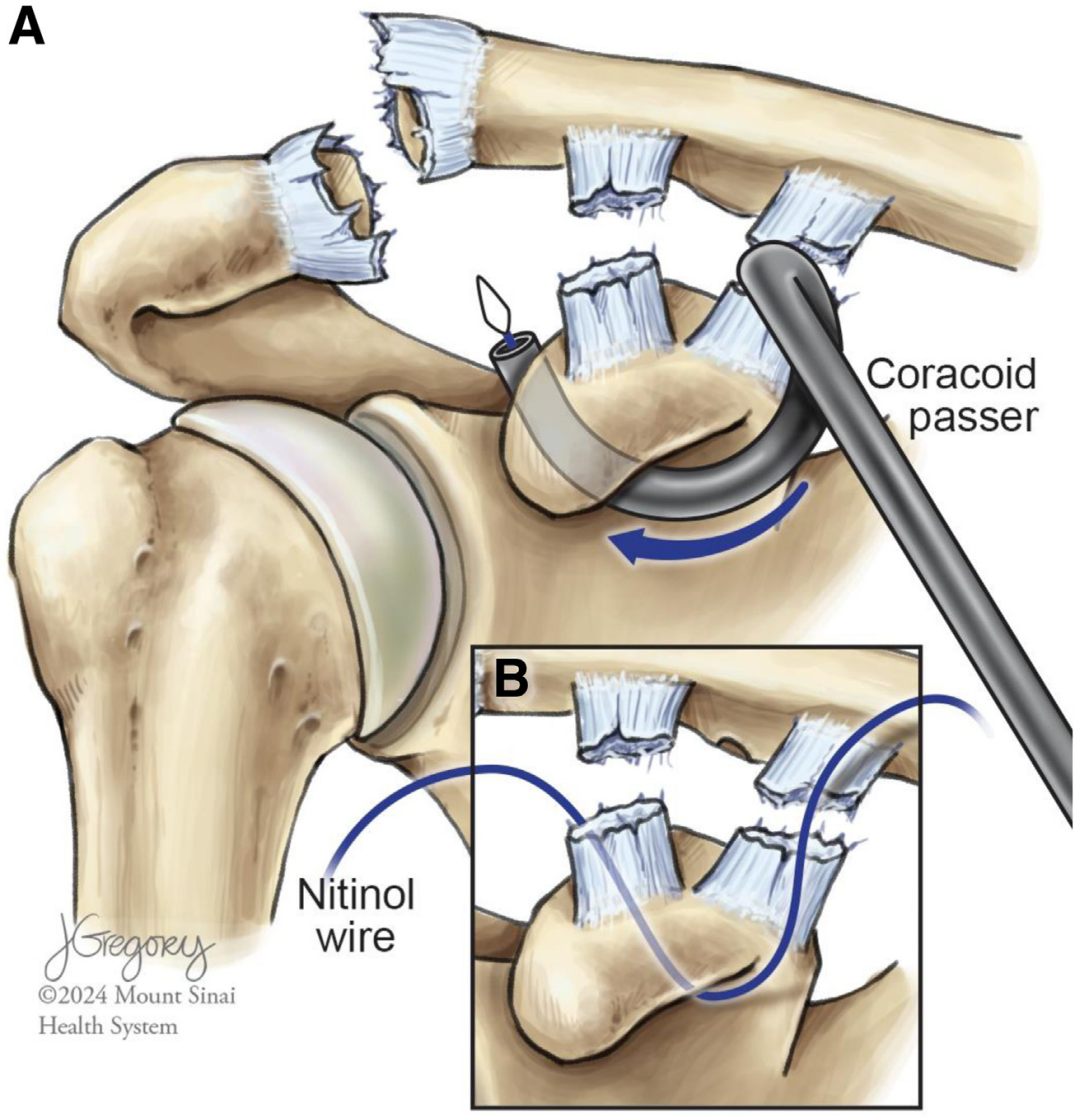
(A) Illustration of a right shoulder with a high-grade acromioclavicular joint separation injury, depicting directionality of the coracoid passer from medial to lateral inferior to the coracoid. (B) Close-up illustration of the path of the nitinol wire inferior to the coracoid after successful passage of the coracoid passer, nitinol wire deployment, and subsequent passer removal.

Using electrocautery, the deltoid and fascia are split vertically. Next, along the anterosuperior surface of the clavicle, 2 cm of deltoid fascia is raised from bone to form a “T-shaped” fascial incision. The coracoid is palpated and the soft tissue overlying its superior surface is split posterior to the origin of the conjoint tendon. A key elevator is introduced and used to elevate the soft tissue circumferentially. A coracoid suture passer loaded with nitinol wire is then placed from medial to lateral along the inferior aspect of the coracoid (Fig 2). Once secured, the nitinol wire is used to shuttle the leading end of the suture tape cerclage (Arthrex) inferior to the coracoid, exiting on its lateral aspect.

**Fig 3:**
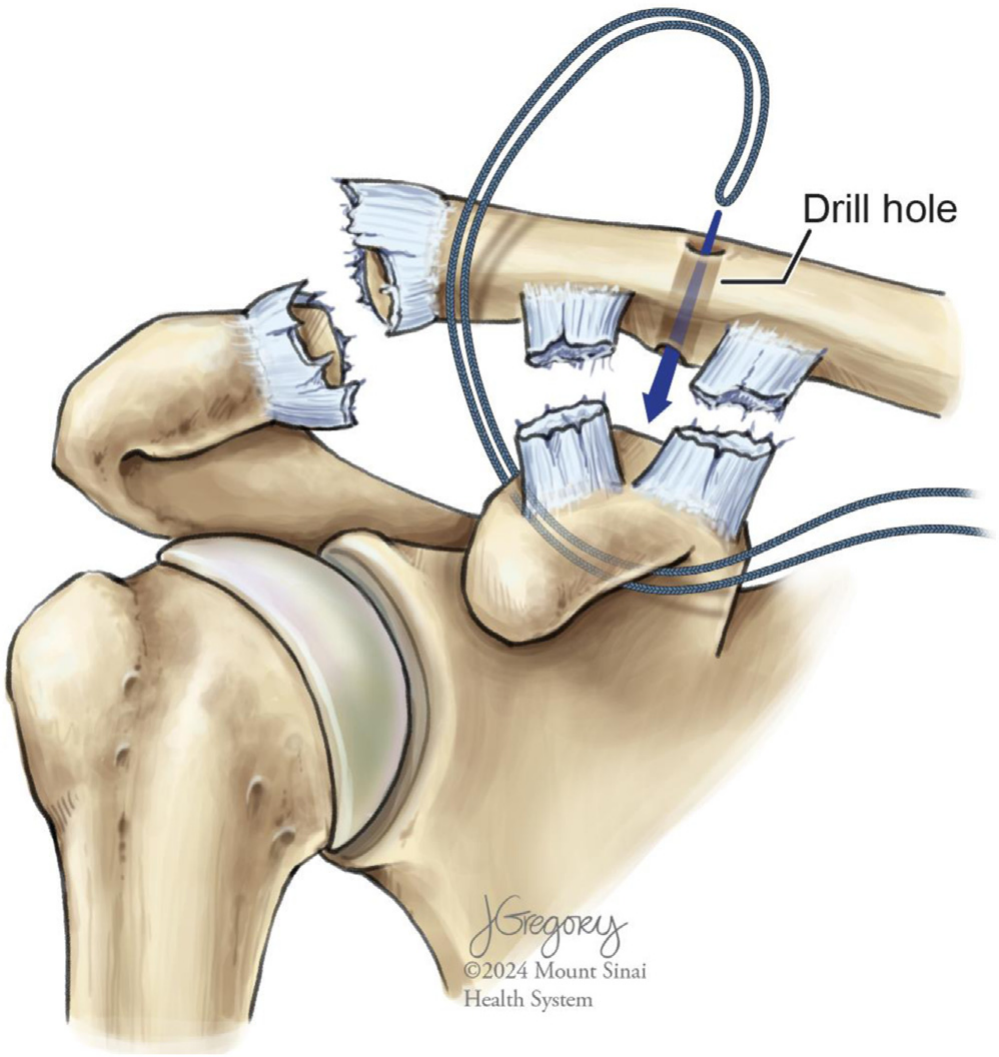
Illustration of a right shoulder with a high-grade acromioclavicular joint separation injury after successfully shuttling the leading end of the suture tape cerclage inferior to the coracoid using the nitinol wire. Note that after traveling from medial to lateral inferior to the coracoid, the leading end of the suture tape cerclage is then passed from superior to inferior through the clavicular drill tunnel.

**Fig 4:**
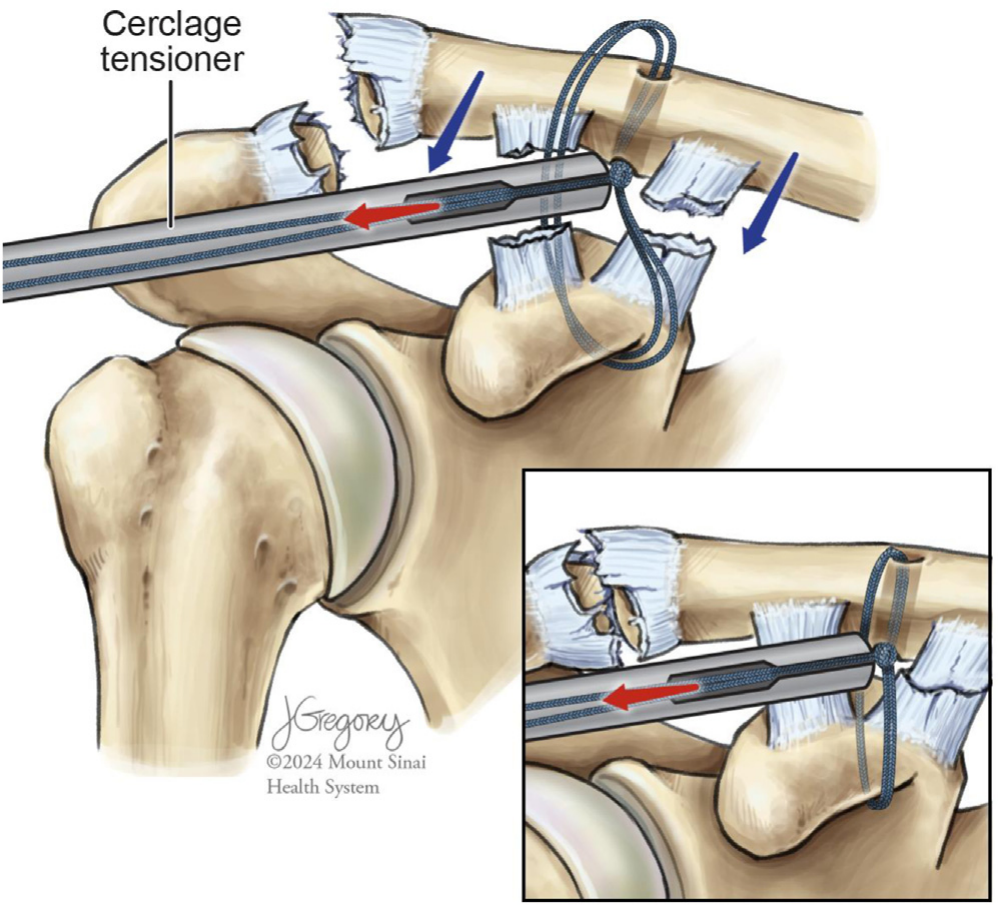
Illustration of a right shoulder with a high-grade acromioclavicular joint separation injury, depicting loading of the suture tape cerclage into the tensioning device after successful passage inferior to the coracoid and through the clavicular drill tunnel. (Inset) Close-up illustration depicting successful reduction of the acromioclavicular joint and coracoclavicular interval upon tensioning of the cerclage with the tensioning device.

To capture the clavicle, a 2-mm drill bit is used to create a tunnel through the clavicle directly superior to the coracoid. The leading edge of the suture tape cerclage is advanced superiorly from its exit on the lateral aspect of the coracoid until it passes the clavicle anteriorly (Fig 3). A suture passer is then used to shuttle the cerclage in a superior-to-inferior direction through the clavicular tunnel. The leading edge of the cerclage is then passed through a pretied knot on the trailing limb of the cerclage. Gross slack is removed and the pretied knot is positioned adjacent to the inferior surface of the clavicle. Both limbs of the suture tape are then fed through the cerclage tensioning device (Arthrex). Increasing tension is applied via the tensioning device until the “80” gauge line is reached (Fig 4).

**Fig 5:**
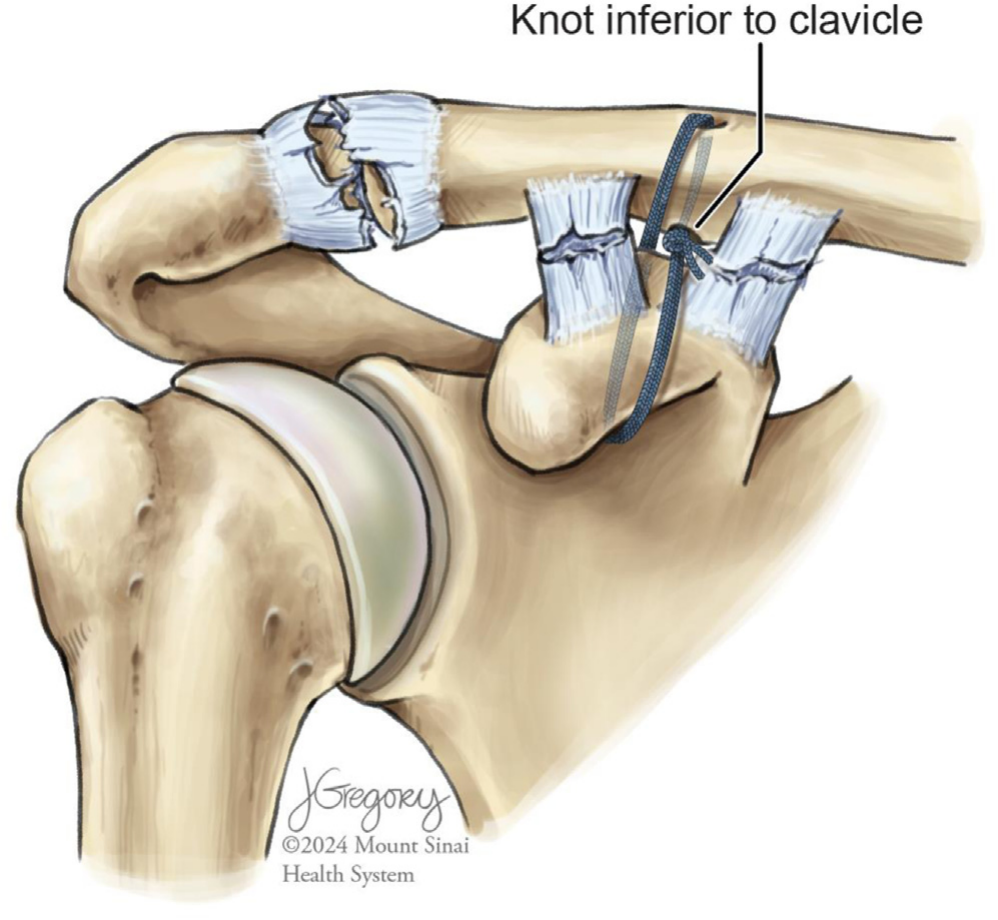
Illustration of a right shoulder, previously with a high-grade acromioclavicular joint separation injury, depicting successful reduction of the acromioclavicular joint and coracoclavicular interval after tensioning the suture tape cerclage. Note that the knot stack is positioned inferior to the clavicle so as to minimize the risk of postoperative hardware irritation.

At this point, with the tensioner in place and still indicating “80,” a mini C-arm is used to evaluate the reduction. After fluoroscopic confirmation, the tensioner is examined. Occasionally, a small amount of slack may spontaneously develop in the system. This is addressed by simply advancing the tensioner back to the “80” mark. The tensioner is released and 3 additional alternating half-hitch knots are thrown to secure the construct. The 2 ends of the cerclage are then once again fed through the tensioner and the tensioner is advanced to the “40” mark to ensure knot security. The 2 limbs of the cerclage are then trimmed (Fig 5).

After thoroughly irrigating the wound, 0 Vicryl is used in figure-of-8 fashion to close the “T-shaped” deltoid and deltoid fascial incision. The overlying soft tissue layers are closed in the usual fashion.

### Technique Modification—Graft Augmentation

In the case of allograft use, the technique proceeds as above up to the point of clearing a path around the coracoid for cerclage passage. When utilizing a graft, the tissue-less channel around the coracoid must be relatively larger to accommodate the increased volume of the graft. To accomplish this, additional pectoralis minor origin medially and coracoacromial ligament laterally must be released from the surface of the coracoid. Carefully, the soft tissue path inferior to the coracoid is expanded with gentle digital pressure.

Once satisfied, the suture tape cerclage and allograft are sequentially shuttled from medial to lateral inferior to the coracoid. The clavicular tunnel is drilled and the cerclage is passed through the clavicle in the superior-to-inferior direction. Electrocautery is then used to release 1 cm of deltotrapezial fascia from the clavicle immediately posterior to the clavicular tunnel. Using a passing stitch, 1 limb of the allograft is advanced from its exit on the lateral aspect of the coracoid and is passed along the posterior aspect of the clavicle in an inferior-to-superior direction. The graft is retrieved and advanced anteriorly over the superior aspect of the clavicle and secured.

As described earlier, the suture tape cerclage is then threaded through the tensioner and the AC joint is reduced. Fluoroscopy is used to confirm reduction. The suture tape is secured with additional half-hitch knots. An Allis clamp is then applied to each end of the allograft and positioned so as to produce graft overlap on the superior surface of the clavicle. Manual tension is applied through the clamps, and the 2 ends of the graft are secured to each other using a No. 2 FiberWire (Arthrex). The excess graft is excised, and the wound is closed in the fashion described above.

### Postoperative Protocol

Postoperatively, patients are placed in a sling and made non–weightbearing in the operative extremity. At 2 weeks, supervised passive range of motion is started. At 6 weeks, the sling is discontinued and active range of motion is initiated. At 10 weeks, strengthening exercises are introduced. Patients are typically cleared for return to all activities at 5 months postoperatively. Further postoperative recommendations are presented in Table 1.

**Table 1:** Postoperative Rehabilitation Protocol After All-Suture Cerclage Fixation of High-Grade Acromioclavicular Joint Separation Injuries

Time (Postoperatively)	Activity Recommendation
0-2 weeks	• Non–weightbearing in sling • No active or passive shoulder range of motion
2-6 weeks	• Non–weightbearing in sling • Passive shoulder range of motion under therapist supervision
6-10 weeks	• Sling discontinued • Protected weightbearing (<5 lbs.) • Active Shoulder Range of Motion
10-20 weeks	• Graduated strengthening exercises
20 weeks	• Return to activity without restriction

## Discussion

In their meta-analysis of 7 separate operative techniques for the treatment of AC joint injuries, Gowd et al.^2^ found suture-based constructs to have the lowest rate of loss of reduction, occurring in 15.1% patients. While promising, this rate of failure remains unacceptably high to most surgeons. Biomechanical study results have suggested that these constructs commonly fail due to compromised suture integrity, leading other groups to investigate the utilization of alternative suture materials.^6^, ^7^, ^8^

Cerclage systems composed of suture tape, a broad, high-strength suture material, were made widely available by industry in 2019 (Arthrex). Although initially marketed for the treatment of periprosthetic humerus fractures, the product was quickly adapted to various orthopaedic procedures.^3^^,^^4^^,^^9^, ^10^, ^11^, ^12^, ^13^, ^14^, ^15^, ^16^ Appreciating early reports of success, investigators began experimenting with suture tape as the primary method of fixation in the treatment of AC joint injuries.

Wellington et al.^1^ recently published a well-designed cadaveric biomechanical study investigating the use of a suture tape cerclage, both alone and with supplementary stabilization, in reconstruction of the CC ligamentous complex. They found no significant difference between the 3 constructs utilizing suture tape but found each of these 3 constructs to be statistically superior in terms of stiffness, displacement, and ultimate load to failure than the lone construct that did not utilize suture tape.

**Fig 6:**
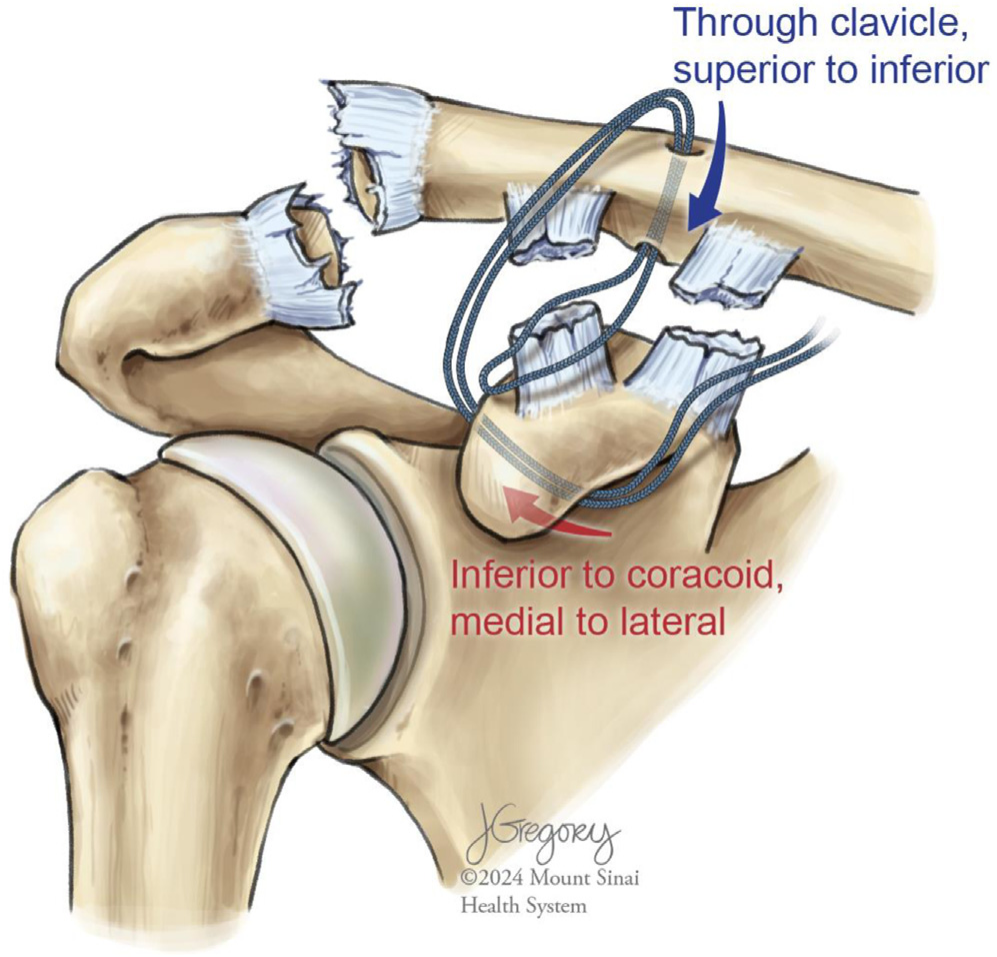
Illustration of a right shoulder with a high-grade acromioclavicular joint separation injury, depicting an overview of the directionality of passage of the suture tape cerclage. The suture tape cerclage is passed from medial to lateral inferior to the coracoid and then from superior to inferior through the clavicular drill tunnel. Correct directionality of passage is critical to appropriate final knot stack placement.

This all-suture tape cerclage technique for the fixation of high-grade AC joint separations was designed to capitalize on the physical properties of this recently developed suture material while minimizing the risk of complications inherent to currently popular procedures. We have found it critical to success that careful attention be paid to the direction of cerclage passage (Fig 6). Pearls and pitfalls of our technique are presented in Table 2 while its advantages and disadvantages are detailed in Table 3.

**Table 2:** Pearls and Pitfalls for Performing the All-Suture Cerclage Fixation Technique in the Fixation of High-Grade Acromioclavicular Joint Separation Injuries

**Pearls**
The utilization of traction stitches, one through the medial and another through the lateral deltoid fascial flaps, allows for gentle retraction and improved coracoid visualization.
Fluoroscopic examination intraoperatively is a useful tool for confirmation of the reduction. Persistent subluxation may indicate inadequate soft tissue clearance along the path of the cerclage.
Allow the system to “relax” after the first tensioning and then recheck the tension measure. Occasionally, a small interval loss of tension will occur due to soft tissue relaxation and/or creep.
**Pitfalls**
Incorrect directionality of cerclage passage will result in the knot stack lying on the superior surface of the clavicle with the potential for symptomatic soft tissue irritation.
Inadequate clearance of soft tissue from bone along the path of the cerclage will lead to the development of slack in the system and partial loss of reduction postoperatively.

**Table 3:** Advantages and Disadvantages of the All-Suture Cerclage Technique for Fixation of High-Grade Acromioclavicular Joint Separation Injuries

**Advantages**
The all-suture construct with a buried knot stack negates the risk of development of postoperative symptomatic hardware.
The absence of a coracoid drill tunnel greatly diminishes the risk of development of iatrogenic coracoid fracture.
Limited material and implant requirements decrease overall cost of procedure.
**Disadvantages**
The use of a clavicular drill tunnel introduces potential risk of development of iatrogenic clavicle fracture.
Indirect reduction of the acromioclavicular joint may result in residual subluxation if intraoperative fluoroscopy is not available.

Despite the myriad described techniques, no reliable gold standard has been established for the operative fixation of high-grade AC joint separation injuries. The surgical technique described in this Technical Note utilizes a state-of-the-art suture material to overcome the limitations of previous constructs and has shown initial promise in the treatment of these notoriously difficult injuries.

## Disclosures

The authors declare the following financial interests/personal relationships which may be considered as potential competing interests: P.J.D. is a consultant or advisor for Arthrex. P.J.C. is a consultant or advisor for Arthrex, Stryker Orthopaedics, Johnson & Johnson Services, and Exactech. All other authors (W.A.R., L.T., A.V.P., C.S., C.M.C.) declare that they have no known competing financial interests or personal relationships that could have appeared to influence the work reported in this paper.

## Supplementary Data

Video 1 Video depicting reconstruction of the coracoclavicular ligament complex utilizing an all-suture tape cerclage technique in a patient with a type V acromioclavicular joint separation injury of the left shoulder. Note the direction of passage—medial to lateral inferior to the coracoid and superior to inferior through the clavicle. This directionality is critical for positioning the knot stack on the inferior surface of the clavicle and effectively eliminating the potential for hardware irritation postoperatively.
